# Fresh raspberry phytochemical extract inhibits hepatic lesion in a Wistar rat model

**DOI:** 10.1186/1743-7075-7-84

**Published:** 2010-11-25

**Authors:** Yan Liu, Ming Liu, Bin Li, Jin-Lu Zhao, Chun-Peng Zhang, Luo-Qiang Lin, Hong-Sheng Chen, Shu-Jun Zhang, Jun-Chao Jin, Lei Wang, Le-Jing Li, Jia-Ren Liu

**Affiliations:** 1 Treatment Center of Oncology, the Fourth Affiliated Hospital of Harbin Medical University, 37 YiYuan Street, NanGang District, Harbin, 150001, The People's Republic of China; 2 The Affiliated Tumor Hospital of Harbin Medical University, 6 BaoJian Road, NanGang District, Harbin, 150086, The People's Republic of China; 3 Public Health College, Harbin Medical University, 157 BaoJian Road, NanGang District, Harbin, 150081, The People's Republic of China; 4 Jia-Ren Liu at Harvard Medical School, 300 Longwood Ave, Boston, MA, USA

## Abstract

**Background:**

Red raspberry possesses potent antioxidant capacity and antiproliferative activity against cancer *in vitro*.

**Methods:**

The objective of this study was to determine the protective effects of raspberry 80% acetone extract in a rat hepatic lesions model induced by diethylnitrosamine (DEN). Rats were treated with the red raspberry extract (0.75, 1.5 or 3.0 g/kg of body weight) by gavage starting 2 h after DEN administration and continuing for 20 weeks.

**Results:**

A dose-dependent inhibition by red raspberry extract of DEN-induced hepatic nodule formation which stands for hepatic lesions was observed. Corresponding hepatic nodule incidence rates were 45.0, 40.0, 25.0 and 5.0% in positive control, low, middle and high groups, respectively (*P *< 0.01 or 0.05). Gross findings, histopathological and ultrastructural evaluations of hepatic lesion were performed on 9, 8, 5 and 1 hepatic nodule in positive control, low, middle and high doses of groups, respectively, identified in rats from the respective groups of 20. A decreasing trend of proportions of hepatocellular carcinoma masses accompanied the increasing doses of red raspberry extract.

**Conclusions:**

These findings demonstrate that the potent capacity of red raspberry diet could not only suppress DEN-induced hepatic lesions in rats, but also reduce the definite diagnostic features of neoplasm.

## Background

Hepatic cancer is a major public health problem throughout the entire world. It was estimated that approximately 24,120 new cases would be diagnosed and more than 18,910 people died from hepatic and intrahepatic bile duct cancers in the United Stated alone in 2010 [1]. Although the incidence rate of hepatic cancer has been falling in most countries worldwide, the decrease has been slower in developing countries than that in the developed countries [2]. By contrast the incidence of primary hepatic cancer is increasing in several developed countries, including the United States [2]. Although diagnosis and treatment are the major strategies of controlling cancer, chemoprevention is one of the best strategies for prevention.

Lifestyle and/or environmental factors, especially dietary factors, play an important role in influencing cancer risk. It has been reported that more than 30% of human cancers could be prevented by an alternative strategy of appropriate dietary modification [3,4]. Epidemiological and laboratory studies, including case-control and cohort studies, have consistently shown that regular consumption of fruits and vegetables is associated with a markedly reduced risk of developing cancer and other chronic diseases [5-12]. Based on the findings that a high consumption of fruit and vegetables is inversely related to a risk of hepatic cancer [11], scientists have suggested that possible anticarcinogenic mechanisms of phytochemicals in fruits and vegetables are primary contributors to the health benefits in the chemoprevention of cancer [13,14]. The health benefits of raspberry or raspberry constituents have been ascribed to: antioxidant [15,16], anti-inflammation [17], low body weight [18], and inhibitory cancer cell growth [19-21]. In previous studies, raspberry extract and its constituents had potent antioxidant capacity, and inhibited the growth of mammary, oral, colon, prostate and liver cancer cells in a dose-dependent manner [19,22]. However, it is not reported that whether raspberry extract inhibits hepatic lesions induced by chemical in an animal model. The objective of the present study was to determine whether raspberry phytochemical extract prevented against hepatic lesions in a rat model.

## Methods

### Red Raspberry Extraction

The red raspberries (*Rubus idaeus *L.) were purchased from a supermarket in China. Fresh red raspberries were cleaned before extraction. The red raspberries were extracted using the method reported previously in our laboratory [9,12]. Briefly, 100 g of fresh weight of the red raspberries were weighed and homogenized with chilled 80% acetone (1:2, w/v) using a chilled Waring blender for 5 min. The sample was then further homogenized using a Polytron homogenizer for an additional 3 min. The homogenates were filtered through Whatman #1 filter paper on a Buchner funnel under vacuum. The filtrate was evaporated at 45°C until approximately 90% of the filtrate had been evaporated. The raspberry extract was standardized to contain 437.62 ± 29.94 mg total phenolics (gallic acid equivalents) per 100 g raspberries. The standardized raspberry extracts were frozen and stored at -80°C until use in the feeding study. Control extracts were also prepared using the same extraction solvents and procedures without red raspberries.

### Animal Care and Treatment

Pathogen-free male Wistar rats, 130 - 140 g of body weight, were purchased from Sino-British SIPPR/BK Lab Animal Ltd (Shanghai, China). The rats adapted immediately to the AIN-93 M diet and were housed in a room with a 12 h light/12 h dark cycle. The rats were acclimated to the surroundings in the animal room for 1 week prior to the initiation of the experiment. Care and treatment of rats followed the recommended guidelines of the National Research Council (1985). The rats were randomly assigned to five groups (n = 20/group). Three raspberry treated and positive control groups of rats were given 10 mg/kg body weight of diethylnitrosamine (DEN, Sigma Chemical Co., St. Louis, MO) water solution by gavage once and continue to drink 0.025% DEN water ad libitum for 20 weeks; the fifth group which received no DEN but was given 1 mL of distilled water served as the negative control group. Rats were administered the control extracts or red raspberry extract starting 2 h after DEN administration and continuing for 20 weeks. Three levels of low, middle, and high doses of red raspberry extract were given to the rats by gavage corresponding to 0.75, 1.5, and 3.0 g fresh raspberry/kg/day body weight raspberry extract, respectively. The control groups fed with control extraction. Animals were weighed weekly. All animals were sacrificed under anesthesia at the end of the 20^th ^week after DEN administration. Livers were rapidly removed and weighed. The relative liver weight was calculated on the basis of final body weights. Livers were cut into 5 to 8 sections to find whether there have nodules which were stand for hepatic lesions of rat induced by DEN.

### Light and Electron Microscope

Part of nodules from the positive control and experimental groups were fixed in 10% neutral formalin solution and subsequently dehydrated and embedded in paraffin. The tissue wax was cut into 5 μm sections, fixed on slides and processed for microscopy examination (stained with hematoxylin and eosin, H&E) analyses. Histopathological evaluation was carried out on coded slides following the IARC (International Agency for Research on Cancer) classification of rodent hepatic lesions [23]. Two independent pathologists evaluated these sections in a double blind manner. Both investigators under a multihead microscope reassessed the sections in cases of disagreement.

For transmission electron microscopy (TEM), portions of nodules were removed by dissection and fixed at 4°C for 1 - 4 h in 3% glutaraldehyde in 0.2% sodium cacodylate buffer (pH 7.0) containing 30 mg/mL NaCl and 2 μg/mL CaCl_2_; postfixation followed at 4°C for 4 h in 1% OsO_4 _minus salts. Sections were rinsed twice in 0.2 mol/L sodium cacodylate buffer and dehydrated through a graded ethanol series. Subsequently, the sections were immersed with Epon 821, embedded in capsules and converged at 60°C for 72 h, and then ultrathin sections (60 nm) were prepared and stained with uranyl acetate and lead citrate. The ultrastructure of cells was examined by TEM [24].

### Statistical Analysis

Data were expressed as mean ± S.D. A one-way analysis of variance was used to analyze body weight. Fisher's Exact Test was used to compare the percentage of rats with liver lesions and histology in each group. Data analyses were generated and plots were constructed using SPSS for Windows version 18.0 (SPSS Inc., Chicago, IL) and SigmaPlot version 11.20 for Windows (Systat Software Inc., San Jose, CA). Statistical significance was set at *P *< 0.05 or *P *< 0.01.

## Results

### Effects of Raspberry Extract on the Prevention of Hepatic Lesions

**Figure 1:**
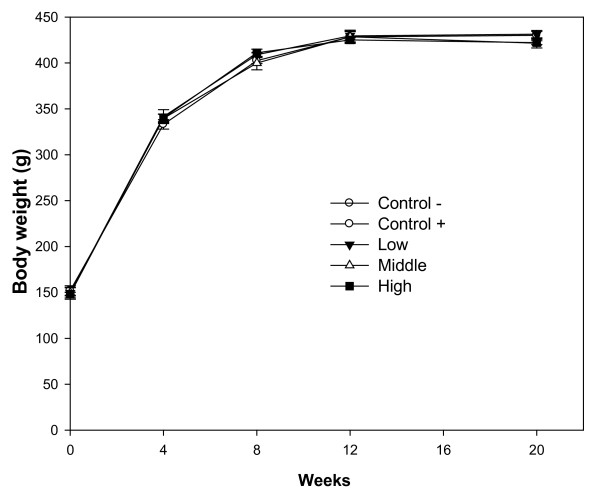
**Body weight**. Wistar rats were fed three levels of 0.75 (low), 1.5 (middle), and 3.0 (high) g of fresh red raspberries/kg of body weight after diethylnitrosamine (DEN) administration and continuing for 20 weeks. Control -, negative control group; control +, positive control group.

Body weights of animals in each group are presented in Figure 1. Throughout the experiment, there were no significant differences in body weight in animals fed raspberry extract and animals in the controls groups (*P *> 0.05). At the termination of the experiment (20 weeks) the relative weights of liver were not significantly different between the negative control and raspberry treated groups (*P *> 0.05) (Table 1). Rats treated with the carcinogen DEN developed hepatic lesions with 45.0% nodule incidence in the positive control group, and 40.0, 25.0, and 5.0% in animals fed the three levels of low, middle, and high doses of raspberry extract, respectively, during the 20-week study. No tumors were detected in the negative control group without DEN (Table 1). Application of low, middle, and high doses of raspberry extract reduced the nodule incidence by 11.1, 44.4, and 88.9% (*P *< 0.05 and *P *< 0.01), respectively, in a dose-dependent manner (Table 1). Total nodule numbers in the control group and the three levels of low, middle, and high dose groups were 9, 8, 5, and 1, respectively, in a dose-dependent manner (Table 1).

**Table 1:** Preventive effects of hepatocarcinogenesis of red raspberry extract in a Wistar rat model ^a^

Group	**no**.	DEN	dose (g/kg of b.w.)	no. of rats with nodules/total no. of rats	nodule incidence (%)	liver index (g/100 g b.w.) (mean ± S.D.)
High	20	+	3.0	1/20	5.0**	2.61 ± 0.49
Middle	20	+	1.5	5/20	25.0*	2.66 ± 0.18
Low	20	+	0.75	8/20	40.0	2.78 ± 0.50
Control -	20	-	-	0/20	0.0	2.90 ± 0.78*
Control +	20	+	-	9/20	45.0	2.54 ± 0.23

a *, *P *< 0.05, **, *P *< 0.01, compared to the corresponding positive control group. Control -, negative control group; control +, positive control group. B.w., body weight.

### Gross findings of rat liver in DEN-induced hepatic lesions

**Figure 2:**
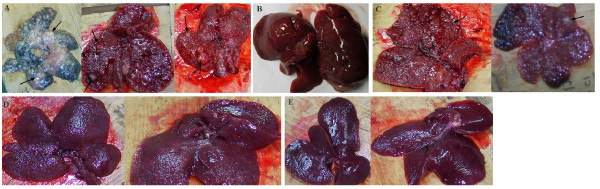
**Gross findings of rat hepatic surface in the raspberry treatments and positive control groups**. The nodules in the hepatic surface were marked by arrows. Grossly visible or tan nodules of hepatic surface were found in the positive control group (A) and low dose of raspberry treated group (C). No nodules in the hepatic surface were found in the negative control group (B), middle (D) and high (E) doses of raspberry treatments.

At the termination of the experiment, the rats were necropsied, and 5 to 8 sections were taken per liver, which enabled us to examine nodules. Nine of twenty rats in the positive control group demonstrated grossly visible, tan nodu1es, which bulged from the external and cut surface of the liver (Figure 2A). The nodules were light gray to yellow-tan or blue and were more friable in consistency than the surrounding normal liver tissue (Figure 2B). These nodules were present uniformly in all lobes and measured from 2 to 8 mm in diameter. Many of the nodules on the liver surface were elevated (Figures 2A and 2C). The nodules in both the low dose of raspberry treatment and positive control groups were sharply demarcated from the surrounding hepatic parenchyma (Figures 2A and 2C). The larger nodules in the positive control group were dull gray, central, soft loci, which upon microscopic examination were seen to be areas of necrosis. No nodules were found in the surface of rat livers in the middle and high doses of raspberry treatments (Figures 2D and 2E). The nodules were evidently fewer in the raspberry treatments than those of the positive control group. The number of rats with hepatic nodules was 8 of 20, 5 of 20, and 1 of 20 rats in the low, middle, and high dose of raspberry treatments, respectively. The livers in the negative control group were soft, pinkish-brown, triangular organs and no nodules were found on the surface or within the livers (Figure 2B).

### Histological Findings

**Figure 3:**
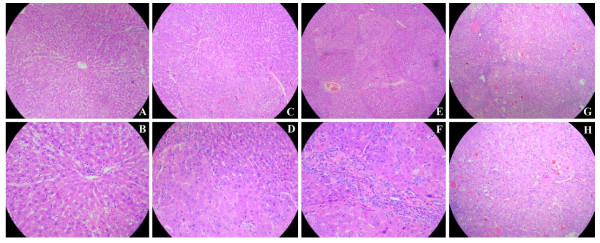
**Histopathological findings in hepatic tumors stained with HE: (**A**, **B**) HE-stained section of a normal hepatic organ in the negative control group (10× and 40×); (**C**, **D**) HE-stained section of a hyperplasia in the high dose group (10× and 40×); (**E**, **F**) HE-stained section of a fibrosis (10× and 40×) in the middle dose group; (**G**, **H**) HE-stained section of a moderately differentiated hepatocellular carcinoma in the positive control group (10× and 40×)**. The neoplastic cells are large, oval- or irregular-shaped, and their cytoplasm is abundant. The nuclei are enlarged, binucleated and pleomorphic nuclei with granular chromatin. Sometimes large basophilic hyperchromatic nuclei are also noticed. The cytoplasm is loose and irregularly extensive vacuolated with masses of acidophilic materials, although this was not invariable.

The nodules taken from rat livers of raspberry treated and positive control groups were examined by histopathology. Histopathological findings of liver sections from various experimental groups of animals are illustrated in Figure 3. Based on the IARC classification of hepatic lesions, there are three major types of histopathological changes of rat hepatic lesions: hyperplasia, fibrosis and hepatocellular adenocarcinoma. In present study, the livers of negative control animals showed normal hepatocellular architecture. Three types of hepatic lesions were found in positive, low, and middle groups. Only one rat with hepatic hyperplasia was found in high dose of raspberry-treated group. The percentages of hepatocellular carcinomas in hepatic tumors in rats treated with the carcinogen DEN were as follows: 55.6% hepatocellular adenocarcinoma in the positive control, and 25.0, 40.0, and 0% in low, middle and high raspberry extract group, respectively. Although there was no significant differences between fibrosis and hyperplasia, increasing trend of hyperplasia and decreasing trend of fibrosis were observed through the raspberry treatment groups from low to high (Table 2). The histological types of hepatic lesion tissues in the positive control and raspberry treated groups are shown in Figure 2 and Table 2.

**Table 2:** Classes of hepatic lesions in the control and red raspberry-treated groups^a^

group	animal (n)	no. of rats with nodules (n)	hyperplasia (n) (%)	fibrosis (n) (%)	hepatocellular carcinoma (n) (%)
High	20	1	1 (100.0)	0 (0.0)	0* (0.0)
Middle	20	5	1 (20.0)	2 (40.0)	2 (40.0)
Low	20	8	2 (25.0)	4 (50.0)	2 (25.0)
Control -	20	0	0 (0.0)	0 (0.0)	0 (0.0)
Control +	20	9	2 (22.2)	2 (22.2)	5 (55.6)

a*, *P *< 0.05, compared to the positive control group (control +).

### Ultrastructural Findings

**Figure 4:**
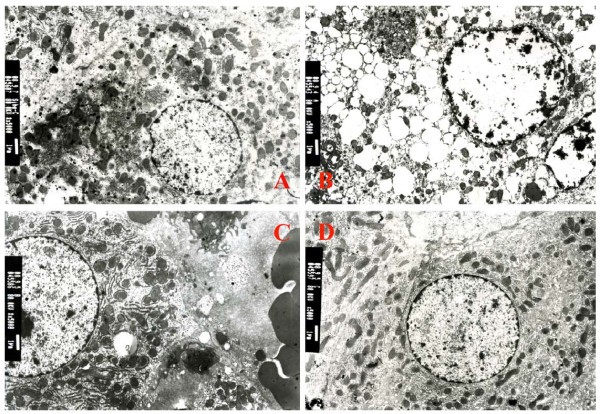
**Transmission electronic microscope findings**. The ultrastructures of normal hepatocyte shown in **A**, and DEN-induced neoplasm cells in groups treated with low (B), middle (C) and high (D) doses raspberry treated groups. The neoplastic cells have an abundant cytoplasm either finely and homogeneously granular, or containing multiple small cytoplasmic vacuoles or some widely scattered hyperchromatic variable-sized nuclei, or rich endoplasmic reticulum, or appearing polyribosomes and monoparticulate glycogen.

Transmission electron microscopy (TEM) was used to determine morphological ultrastructure of hepatic tissue sections. The ultrastructural findings of hepatic tissues of rats treated with red raspberry extract or controls are shown in Figure 4. Epon-embedded sections of hepatic tissue showed regular cellular membrane, organelle, and clear nuclear structure (Figure 4A). The neoplastic cells had an abundant cytoplasm either finely and homogeneously granular or containing multiple small cytoplasmic vacuoles and some widely scattered hyperchromatic variable-sized nuclei (Figure 4B). Nuclear inclusion appeared, rough endoplasmic reticulum degranulated and vacuoles formed in the cytoplasm (Figure 4B). Many ribosomes were scattered throughout the cytoplasm and these often formed polysomes (Figures 4A, 4C and 4D). Mitochondria were largely granular matrix (Figure 4C). Rough endoplasmic reticulum in parallel or nonparallel array was a common cytoplasmic organelle (Figures 4C and 4D). Numerous densely osmiophilic, irregularly shaped, diminutive electron-opaque cytoplasmic structures often lacking both a discernible substructure and definite diagnostic features were consistent with the appearance of lysosomes (Figures 4C and 4D). In this study, DEN-induced hepatic neoplasm of rat in the positive control group showed the ultrastructural changes such as rich endoplasmic reticulum, appearing polyribosomes and monoparticulate glycogen, widely scattered hyperchromatic variable-sized nuclei. However, these changes were lessened in the raspberry treatments. Thus, red raspberry diet could not only reduce nodule incidence, but also reduce markers of definite diagnostic features of neoplasm.

## Discussion

Red raspberry has been used throughout the centuries for nutritional and medicinal purposes. In a previous study, the red raspberry extract showed a significant inhibition of growth of human oral (KB, CAL-27), breast (MCF-7), colon (HT-29, HCT116), and prostate (LNCaP) tumor cell lines at doses ranging from 25 to 200 μg/mL and stimulation of apoptosis of the Cox-2 expressing colon cancer cell line, HT-29 [19]. Liu et al. also showed that four varieties of raspberry significantly inhibited the proliferation of HepG_2 _human liver cancer cells in a dose-dependent manner [22]. In another study, red raspberry extract significantly inhibited the proliferation of human hepatic cancer HepG2 and human colon cancer CaCo-2 cells in a dose-dependent manner [22,25]. The red raspberry extract was also shown to inhibit cell proliferation of HT-29 colon cancer mainly via the p21^WAF1 ^pathway [26]. The red raspberry extract and its components reduced endogenous oxidative DNA damage *in vitro *and *in vivo *[27].

Diethylnitrosamine (DEN) is a representative chemical carcinogen with the potential to cause tumors in various organs, including the liver, skin, gastrointestinal tract, and respiratory system. DEN is a complete carcinogen to induce hepatocellular carcinoma [28]. DEN is also well known as a reliable carcinogen to induce lesions in rat that mimic the different types of benign and malignant tumors in human [29,30]. In this study, rats were fed three levels of 0.75 (low), 1.5 (middle), and 3.0 (high) g fresh red raspberries/kg body weight per day starting after DEN administration and continuing for 20 weeks. The positive control group with carcinogen DEN developed hepatic lesions with 45.0% nodule incidence and no nodules were detected in the negative control group without DEN. A dose-dependent inhibition of hepatic lesions by fresh red raspberry extract was observed (Table 1). These results demonstrate for the first time that red raspberry extract has potent anticarcinogenic activity induced by DEN in a rat model. The nodule incidence was decreased in a dose-dependent manner. This study also first reported the incidence of hepatic lesion in a DEN-induced rat model.

At the termination of the experiment, the rats were necropsied, and grossly visible, tan nodu1es, which bulged from the external and cut surface of the liver, were found in the positive and red raspberry treated groups. These nodules were analyzed by histopathological staining. The main histopathological differential diagnoses in the hepatic lesion tissues include hyperplasia, fibrosis and hepatocellular carcinomas based on the *IARC *classification of rodent tumors [23]. The normal liver shows normal hepatocellular architecture mainly consisting of normal hepatic parenchyma (Figure 3A and 3B). The hepatic hyperplasia shows cell proliferation and little difference from those in normal liver and the cellular architecture of hepatocyte presented almost normal hepatocellular architecture (Figures 3C and 3D). The hepatic fibrosis shows that nodules were also variably encased in fibroconnective tissue, which in turn was invested by a rim of compressed hepatocytes (Figures 3E and 3F). The hepatocellular carcinoma shows that within the nodular areas, bile ducts were scanty or absent and lobular architecture was not preserved. The individual intranodular hepatocytes were large, oval- or irregular-shaped, and their cytoplasm was abundant. The cytoplasm was loose and irregularly extensive vacuolated with masses of acidophilic materials, although this was not invariable (Figures 3G and 3H). The nucleus was often binucleated and pleomorphic nuclei with granular chromatin. Sometimes large basophilic hyperchromatic nuclei were also found (Figure 3H). The type of hepatocellular carcinomas is a kind of highly malignant tumor and also the main cause of death in hepatic cancer animals and hepatic cancer patients, and is a main target for chemotherapy. Hepatocellular carcinomas are always accompanied by occurrence of distant metastasis and areas of necrosis, ulceration, and hemorrhage [31,32]. Some phytochemicals, including curcumin, resveratrol, green tea catechins, oltipraz and silibinin shown to suppress hepatic carcinogenesis in experimental studies, possess promising chemopreventive and chemotherapeutic properties [33,34]. These phytochemicals will bring wide perspectives for liver cancer patients, specifically those with hepatocellular carcinoma. Any potential agents are generally tolerated, nontoxic, inexpensive and sufficiently bioavailable, and acceptable to patients by oral administration. In our study, there was no toxicity observed in the animals fed the red raspberry extract at the doses tested. The pathological findings in this study showed that there was 55.6% hepatocellular adenocarcinoma in the control group and 25.0, 40.0, and 0% in the three levels of low, middle, and high doses of red raspberry treatment, respectively, during the 20-week study (*P *< 0.05). The ultrastructural findings showed that the neoplastic cells had an abundant cytoplasm either finely and homogeneously granular, or containing multiple small cytoplasmic vacuoles or some widely scattered hyperchromatic variable-sized nuclei, or rich endoplasmic reticulum, or appearing polyribosomes and monoparticulate glycogen. Thus, red raspberry diet could not only reduce nodule incidence, but also reduce markers of the diagnostic features of the neoplasm. Red raspberry might be a potential candidate agent of chemoprevention or therapeutics for hepatocellular adenocarcinoma.

The major groups of phenolic compounds present in red raspberries are reported to be anthocyanins, flavonols, flavanols, ellagitannins, gallotannins, proanthocyanidins, and phenolic acids [35]. The major anthocyanins and tannins identified are as follows: cyanidin-3-sophoroside, cyanidin-3-glucoside, pelargonidin-3-glucoside and predominantly hydrolyzable tannins (ellagitannins and gallotannins) as well as ellagic and gallic acid. A mixture of 15 anthocyanidin 3-*O*-glycosides, cyaniding 3-glucoside, ellagic acid, and gallic acid decreased the proliferation of HT-29 cells in a dose - dependent manner [26]. These compounds are metabolized by the liver, implying the liver is directly exposed to these compounds by compounds themselves or metabolites, which may explain why the raspberry diet so effectively protected the liver lesions against a DEN insult. However, we did not analyze the phytochemical composition of this extraction. The constituents of the red raspberry extract used in this study as well as their bioavailability need to be further analyzed.

## Conclusion

In summary, our data suggested that fresh red raspberry extract possesses potent activity to suppress DEN-induced hepatic lesions in a rat model. The extracts not only inhibited incidence of nodules, but also suppressed the ratio of hepatocellular adenoma and reduced the definite diagnostic features of neoplasm in a DEN-induced rat model. However, the exact mechanism(s) of how red raspberries prevent hepatic lesions needs to be further studied.

## Competing interests

The authors declare that they have no competing interests.

## Authors' contributions

YL, ML and BL contributed equally to the study i.e. experimental design, feeding animal, getting tissue samples and analyzing data of this manuscript. ZJL, ZCP and LLQ have participated in feeding animal and sample preparation of the study. CHS, ZSJ, JJC, WL and LLJ contributed to the hepatic pathology and reviewing HE slides as well as TEM interpretation. JRL and ML analyzed and interpreted the data, drafted and critical revised the manuscript, and approved the final version of the article. All authors have read and approved the final manuscript.
